# Experience of Vaginoplasty for Enhancement of Sexual Functioning in a Center in Turkey: A Before and After Study

**DOI:** 10.7759/cureus.14767

**Published:** 2021-04-30

**Authors:** Gokcen Erdogan

**Affiliations:** 1 Gynecology and Obstetrics, Near East University Medical Faculty, Nicosia, CYP

**Keywords:** sexual dysfunction, sexual satisfaction, vaginoplasty, golombok rust inventory of sexual satisfaction

## Abstract

Objective

The number of vaginal rejuvenation procedures for improvement of sexual function is dramatically increasing worldwide. The objective of this study was to present our experience with women who presented to our clinic with the complaint of sexual dysfunction or desire to enhance sexual function or orgasm.

Methods

Demographic and descriptive data of the patients were evaluated. In addition, sexual dysfunction of the patients who underwent vaginoplasty in our center were evaluated before and after vaginoplasty procedure using Golombok Rust Inventory of Sexual Satisfaction (GRISS) scale and the scores were compared before and after the procedure, which is used in the evaluation of sexual dysfunction by relationship counsellors and clinics.

Results

A total of 250 women who described a sensation of a wide or floppy vagina with lost or decreased ability to achieve orgasm were included in the study. The mean age of the patients was 38.51±9.126 years. Of all women, 85.2% were college graduates. A history of normal vaginal delivery was found in 77.8% of the participants. The mean GRISS scores of “Infrequency”, “Non-communication”, “Dissatisfaction”, “Non-sensuality”, “Avoidance”, “Anorgasmia” and “overall GRISS” scores were statistically significantly decreased, while the mean vaginismus score was significantly increased (p<0.01).

Conclusion

Highly satisfying outcomes regarding patient satisfaction were obtained from vaginoplasty procedures that we have performed.

## Introduction

Sexuality and sexual activity are important determinants of quality of life (QoL) and life satisfaction. Sexual satisfaction perceived individually by each person is a crucial component of sexual activity. Sexual health focuses not only on treatment, but also on achieving the highest level of well-being, QoL and sexual pleasure or satisfaction as possible. Female sexual functioning is influenced by previous vaginal delivery, ageing, genetic factors, relationships with the partner and other psychosocial factors. These factors may cause several disorders ranging from sexual dissatisfaction to actual deviation.

After vaginal trauma, most commonly vaginal delivery, women may encounter vaginal laxity due to stretching of local tissue and separation of the pelvic floor musculature. Postpartum vaginal laxity causes a gaping perineum, decreasing sensation of friction and sexual satisfaction [1]. There is abundant epidemiological evidence that vaginal delivery is the strongest factor of pelvic floor disorders [2]. Women with a history of vaginal delivery, and especially multiparous women may complain of alterations in sexual satisfaction, decreased friction during intercourse, changes in vaginal sensation and a general feeling of laxity [3-5]. In addition, women usually complain of the gaping of the vaginal vestibule that causes the appearance of vaginal mucosa [6]. This gaping leads to several functional concerns such as increased vaginal secretions due to mucosa exposure, alterations in achieving orgasm and vaginal air entrapment, which causes embarrassing sounds during intercourse [7].

Surgical vaginal tightening procedures are not new, although historically these procedures have been performed for obstetric postpartum repair rather than sexual and esthetic concerns [2]. Today, these procedures have increasingly gained popularity, although the line between enhancement of sexual functioning and medically indicated surgical procedures is a grey area and vaginal tightening procedures are performed for both purposes. The number of vaginal rejuvenation procedures for improvement of sexual function is dramatically increasing worldwide [8]. A few studies in the literature have shown that vaginal tightening procedures including vaginoplasty and perineoplasty are associated with enhanced sexual function with low rates of complications [1,5]. The reported minor complication rates vary between 3.8% and 19.7% [9].

The objective of this study was to retrospectively investigate the changes between pre- and post-vaginoplasty sexual functioning parameters and to share our experience with women who presented to our clinic with the desire of enhancing sexual function or pleasure.

## Materials and methods

A total of 250 female patients who presented to our clinic and underwent vaginoplasty operation between January 2018 and April 2020 were retrospectively evaluated in the study. A detailed history was received from all patients at the time of admission and physical examinations were performed. The physical examination was performed both while a patient bears down and was in the lithotomy position, and the vaginal width and levator ani muscles were assessed [10]. After physical examination, patients were informed about vaginoplasty procedure, possible outcomes, complications and postoperative care, and gave written consent for the operation.

Patients using drugs affecting sexual function, those with symptomatic prolapsus (cystocele), rectocele or uterine prolapsus, patients with dyspareunia, primary anorgasmia or any psychological disorder (all patients were psychologically evaluated before the surgery), those with partner’s sexual dysfunction, a history of pelvic surgery, those receiving radiotherapy for vaginal or cervical cancer, patients with chronic vaginal infections, those without intact pelvic floor support and who denied participation were excluded from the study.

Patients’ demographic characteristics, educational status, childbearing status, number of children, history of vaginal delivery and cesarean section, and numbers of normal delivery and caesarean section were recorded and analyzed. In addition, sexual dysfunction of the patients was evaluated before and after vaginoplasty procedure using Golombok Rust Inventory of Sexual Satisfaction (GRISS) scale and GRISS scores were compared between before and after the procedure.

Surgical technique

Vaginoplasty procedure was performed following the procedure described by Moore et al. [11]. Accordingly, vaginoplasty procedure included the repair of the posterior vaginal canal and introitus and the modification of final diameter and caliber of the vagina to return its prenatal condition. Vaginoplasty procedure was performed under local anesthesia in some patients and under general anesthesia in the other patients who rejected local anesthesia. General anesthesia was performed under operating room conditions. Propofol, rocuronium bromide and fentanyl were used to induce anesthesia. The patients were intubated using laryngeal masks, airway or endotracheal tubes. Remifentanil was used for the maintenance of general anesthesia.

Since local anesthesia offers a reproducible, highly safe and relatively longer-duration anesthesia depending on the anesthetic agent used; local incisional anesthesia was chosen for vaginal esthetics procedures, and bupivacaine with 0.5% epinephrine was preferred as the anesthetic agent. During this anesthetic technique, both intravascular injection was avoided and attention was paid to not exceed toxic doses.

At the beginning of the procedure, inferior edges of the labia majora that will form the posterior fourchette of the vaginal opening were marked to create vaginal introitus during the closure. First, a typical trapeze-shape incision that will also be used in the perineoplasty part of the procedure was made in the introitus. A small incision was then made in the posterior wall and the vaginal epithelium was laterally dissected from the underlying rectovaginal fascia out of the levators. The dissection was continued up to the apex so as to involve the entire posterior wall. The dissection of the vaginal epithelium was performed using Metzenbaum scissors and No. 20 scalpel. The vaginal caliber was then addressed by plication of the rectovaginal fascia with delayed absorbable sutures in the midline. Diameter of the vagina was continuously measured with fingers. A small amount of vaginal epithelium was then excised and the incision was closed with a running fashion. An appropriate amount of skin was excised from the perineum and introitus to provide an aesthetically satisfying appearance of vaginal opening and the procedure was completed with a multilayer repair. For the closure, figure-of-eight 2-0 Monocryl sutures approximated the levators. The superficial transverse perineal muscles and bulbocavernosus muscles were similarly approximated. The vaginal mucosa was closed using 2-0 running Vicryl, and the skin using 4-0 chromic suture. 

Golombok Rust Inventory of Sexual Satisfaction (GRISS)

GRISS is a 28-items questionnaire used in the evaluation of sexual dysfunction in heterosexual couples. Each item is scored with a 5-point Likert scale (i.e. always to never). Raw points obtained from the scale are transformed to standard points between 0 and 9.

GRISS is used by relationship counsellors and clinics in order to detect and follow-up sexual problems. This questionnaire has also been used in clinical trials in which new treatment approaches were applied. GRISS has two versions as female and male.

GRISS involves seven subscales with four questions in each. These subscales include anorgasmia, vaginismus, non-communication, infrequency, avoidance, non-sensuality and dissatisfaction. Participants were asked to fill the questionnaire form before vaginoplasty procedure and during control visit six months after the procedure. Filling of each form lasted for about 15 minutes. The questionnaire was developed by Rust and Golombok [12] and adopted to Turkish by Tugrul et al. [13], and evidence was obtained for reliability and validity of the questionnaire. In Turkish adaptation of GRISS, Cronbach alpha coefficient was found as 0.91 for overall score [13].

Ethics considerations

Before the beginning, the study protocol was approved by the local ethics committee of our hospital (Date:03/05/2020, No:003). The patients were informed about the objectives of the study and gave written consent. The study was conducted in accordance with the ethical principles of the Declaration of Helsinki.

Statistical analysis

Data obtained in this study were statistically analyzed using IBM SPSS version 23 statistical package software (SPSS, Statistical Package for Social Sciences, IBM Inc, Chicago, IL, USA). When evaluating study data, categorical variables are expressed as number and percentage, and numerical variables with mean ± standard deviation among the descriptive statistics. Coefficient of skewness and kurtosis were examined for normality assumption of the numerical variables and the coefficients were found to be within the range of ± 1.5. Therefore, parametric statistical methods were utilized in the analysis. Differences between two numerical variables (e.g. scale points before and after the procedure) were analyzed using a dependent sample t-test. p<0.05 values were considered statistically significant.

Cronbach alpha coefficient of GRISS was calculated as 0.704 for pre-vaginoplasty and 0.836 for post-vaginoplasty evaluations.

## Results

A total of 250 patients who presented to our clinic with the desire of enhancing sexual functioning and underwent vaginoplasty between January 2018 and April 2020 were included in the study. The mean age of the patients was 38.51±9.126 years. Of all women 41 (16.4%) aged over 50 years. When educational status of the participants was examined; 213 (85.2%) of the women were college graduates.

Only two (0.8%) participants had no children. Childbearing women most commonly had two children (59.3%). A history of normal vaginal delivery was found in 193 (77.8%) women, while a history of cesarean section was found in 153 (61.2%) women. Demographic features and descriptive characteristics of the participants are given in Table 1.

**Table 1:** Descriptive characteristics of the participants.

(n=250)	Number	%
Age (mean=38.51; SD=9.126)		
22-30 years	59	23.6
31-39 years	80	32.0
40-49 years	70	28.0
50 years and above	41	16.4
Educational status		
Middle school	4	1.6
High school	33	13.2
College	213	85.2
Childbearing		
Yes	248	99.2
No	2	0.8
Number of Children (n=248)		
1	46	18.5
2	147	59.3
3	48	19.4
4	7	2.8
Normal vaginal delivery		
Yes	193	77.8
No	55	22.2
Number of normal vaginal deliveries (n=193)		
1	104	53.9
2	63	32.6
3	23	11.9
4	3	1.6
Cesarean section		
Yes	153	61.2
No	97	38.8
Number of cesarean sections (n=153)		
1	105	68.6
2	48	31.4

Sexual dysfunction and satisfaction of the participants were measured using GRISS scale. Accordingly, the mean infrequency score of the women was 6.18±1.072 before the procedure, while this score was decreased to 3.30±1.775 after the procedure. The mean non-communication score was 7.64±1.072 before vaginoplasty procedure and 5.93±1.598 after vaginoplasty procedure. The mean dissatisfaction score was 6.58±0.903 before the procedure, while this score was found as 4.24±1.319 after the procedure. The mean non-sensuality score was 8.48±0.718 before vaginoplasty procedure and 5.93±1.473 after the procedure. The mean vaginismus score was 4.81±0.741 before the procedure, while this score was calculated as 5.91±0.778 after the procedure. The mean anorgasmia score was 5.38±0.803 before the intervention, while this score dropped to 3.99±0.946 after the intervention.

The mean overall GRISS score was calculated as 7.32±0.724 before vaginoplasty procedure and 4.52±1.457 after vaginoplasty procedure. As a result of the dependent sample t-test; the mean scores of “Infrequency”, “Non-communication”, “Dissatisfaction”, “Non-sensuality”, “Avoidance”, “Vaginismus”, “Anorgasmia” and “GRISS” show statistically significant differences (p<0.05). The mean infrequency, non-communication, dissatisfaction, avoidance, non-sensuality, anorgasmi and overall GRISS scores were significantly decreased, while the mean vaginismus score was significantly increased (Table 2).

**Table 2:** Differences in Golombok Rust Inventory of Sexual Satisfaction (GRISS) and subscale scores before and after vaginoplasty. *p<0.05 (statistically significant).  t: dependent sample t-test.

	Before vaginoplasty		After vaginoplasty		t	p
	Mean	SD	Mean	SD		
Infrequency	6.18	1.072	3.30	1.775	26.984	0.000*
Non-communication	7.64	0.964	5.93	1.595	14.551	0.000*
Dissatisfaction	6.58	0.903	4.24	1.319	35.790	0.000*
Avoidance	7.29	0.864	5.06	1.261	24.646	0.000*
Non-sensuality	8.48	0.718	5.93	1.470	31.578	0.000*
Vaginismus	4.81	0.741	5.91	0.778	-18.567	0.000*
Anorgasmia	5.38	0.803	3.99	0.946	27.606	0.000*
Total	7.32	0.724	4.52	1.457	39.146	0.000*

**Figure 1:**
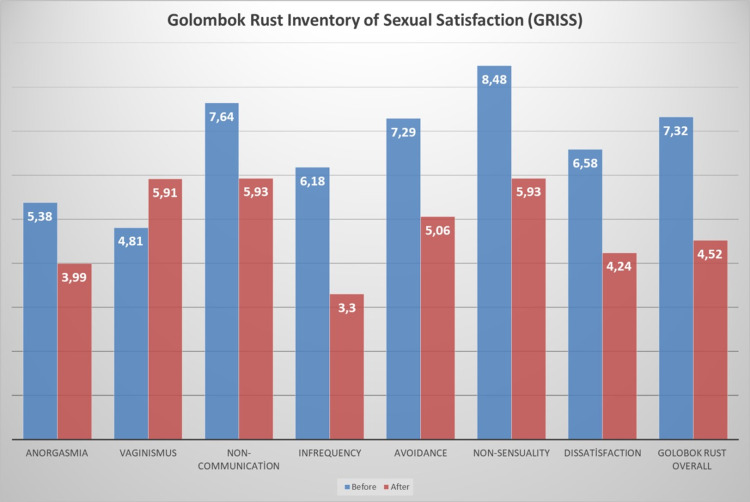
Changes in GRISS subscales before and after vaginoplasty procedure.

Changes in the scores according to the subscales of GRISS scale are shown in Figure 1.

## Discussion

Vaginoplasty and vaginal tightening procedures have become increasingly popular for cosmetic reasons, enhancement of sexual function, treatment of vaginal laxity and urinary incontinence, although scientific short-term and long-term evidence are lacking. Today, the most common indication for vaginoplasty performed to improve sexual function is sexual dysfunction due to vaginal laxity caused by normal vaginal delivery and natural ageing process. According to a survey conducted by the International Urogynecological Association in 2012, 84% of physicians believe that vaginal laxity is underreported and 95% think that laxity affects sexual function [14]. In addition, 40% of women have psychological distress resulted from sexual dysfunction, although only 14% of women counsel to a physician about sex during their lifetime [15]. As knowledge of patients on treatment options for vaginal laxity increases, demand for vaginoplasty will continue to grow. Today taboos on the communication about female dysfunction have broken owing to public awareness programs, physician education and media. Information about these conditions and their treatment are more readily available, increasing the demand for these procedures. In a study from India, demand for esthetic vaginal procedure has risen to 28.92% in 2015 from 3.9% in 2012 [16]. In a review of vaginal tightening procedures performed between 2014 and 2019 in Canada, Austin et al. reported that posterior vaginoplasty resulted in a high patient satisfaction without complications [17]. In our study, satisfying results were obtained in terms of patient satisfaction for enhancement of sexual functioning in patients who underwent vaginoplasty due to sexual dysfunction. The mean overall GRISS score was 7.32±0.724 at the beginning of the study, while this score was decreased to 4.52±1.457 after vaginoplasty procedure.

Information on vaginal rejuvenation procedures mostly comes from the Internet and scientific literature on this issue is very limited. The number of studies in the literature evaluating vaginoplasty performed for enhancement of sexual function is limited and these studies usually include specific patient groups and case reports [3,18,19]. In addition, numerous tools are used in order to measure satisfaction of patients undergoing vaginoplasty. This makes an exact comparison between our results and those reported in the literature difficult.

In a study by Austin et al., investigating patients who underwent vaginoplasty procedure, the mean age of the patient was found as 41 years [17]. In the present study, the mean age of the patients was found as 38.51±9.126 years. The mean age of our patients was consistent with the literature.

In the study by Austin et al., majority of the patients (86.7%) undergoing vaginoplasty were multiparous, while the remaining 13.3% patients were primiparous [17]. In our study, 80.8% of the participants were multiparous, while the remaining 19.2% patients were primiparous. These results are similar to the previously reported rates.

In a study by Crouch et al. in which outcomes of vaginoplasty procedures performed in women with congenital adrenal hyperplasia with GRISS, women who underwent vaginoplasty continued to complain of clitoris sensitivity, vaginal penetration difficulties and frequency of intercourse [20]. In our study, frequency of intercourse was increased by 87.27% after the procedure compared to before the procedure according to the GRISS scale.

In a multicenter cross-sectional study, 47 women underwent vaginoplasty/perineoplasty procedures and the outcomes showed a high satisfaction rate both for women and their partners in terms of the enhancement of sexual function [3]. In our study, the mean “dissatisfaction” subscale score was 6.58±0.903 before vaginoplasty procedure, while this score dropped to 4.24±1.319 after the procedure. In other words, sexual satisfaction was increased by 55.99% after the procedure.

When other findings of our study were examined; communication was increased by 28.84%, sensuality by 43.00%, achieving orgasm by 34.85% after the procedure compared to before the procedure, while avoidance was decreased by 44.07% and vaginismus by 22.87%. These results indicated that our vaginoplasty applications were successful and provided satisfying outcomes for the patients. As mentioned before, because publications in the literature on this issue were lacking, our results could not be exactly compared with the results of other studies.

To our very knowledge, this study is the first in the literature to evaluate satisfaction of the patients undergoing vaginoplasty for enhancement of sexual functioning using GRISS scale. The results of our study will be guiding for further multicenter and comprehensive studies to be performed in future.

Study limitations

The main limitation of our study was lack of an exact comparison of the results with previous studies in the literature. In addition, the results could be compared with a control group. However, the number of our patients was relatively high for such a study. Finally, as a strength aspect this study is the first in the literature on this issue.

## Conclusions

Today, demand for vaginoplasty procedures is increasing due to medical problems as well as cosmetic reasons and desire to enhance sexual function. Highly satisfying outcomes regarding patient satisfaction were obtained from vaginoplasty procedures performed in our center. However, the number of studies on this issue are very limited. Further studies with large series are needed in order to better evaluate outcomes of these relatively new procedures and to shed light on future projections.
